# Puerperal sepsis, the leading cause of maternal deaths at a Tertiary University Teaching Hospital in Uganda

**DOI:** 10.1186/s12884-016-0986-9

**Published:** 2016-08-05

**Authors:** Joseph Ngonzi, Yarine Fajardo Tornes, Peter Kivunike Mukasa, Wasswa Salongo, Jerome Kabakyenga, Masembe Sezalio, Kristien Wouters, Yves Jacqueym, Jean-Pierre Van Geertruyden

**Affiliations:** 1 Department of Obstetrics and Gynecology, Mbarara University of Science and Technology, Mbarara, Uganda; 2 Mbarara University of Science and Technology, Institute of Maternal Newborn Health, Mbarara, Uganda; 3 Department of Obstetrics and Gynecology, Antwerp University Hospital, Antwerp, Belgium; 4 International Health Unit Department of Epidemiology, University of Antwerp, Antwerp, Belgium

**Keywords:** Maternal mortality, Puerperal sepsis, Mbarara University, Obstetrical hemorrhage, Uganda

## Abstract

**Background:**

Maternal mortality is highest in sub-Saharan Africa. In Uganda, the WHO- MDG 5 (aimed at reducing maternal mortality by 75 % between 1990 and 2015) has not been attained. The current maternal mortality ratio (MMR) in Uganda is 438 per 100,000 live births coming from 550 per 100,000 in 1990. This study sets out to find causes and predictors of maternal deaths in a tertiary University teaching Hospital in Uganda.

**Methods:**

The study was a retrospective unmatched case control study which was carried out at the maternity unit of Mbarara Regional Referral Hospital (MRRH). The sample included pregnant women aged 15–49 years admitted to the Maternity unit between January 2011 and November 2014. Data from patient charts of 139 maternal deaths (cases) and 417 controls was collected using a standard audit/data extraction form. Multivariable logistic regression analysis was used to assess for the factors associated with maternal mortality.

**Results:**

Direct causes of mortality accounted for 77.7 % while indirect causes contributed 22.3 %. The most frequent cause of maternal mortality was puerperal sepsis (30.9 %), followed by obstetric hemorrhage (21.6 %), hypertensive disorders in pregnancy (14.4 %), abortion complications (10.8 %). Malaria was the commonest indirect cause of mortality accounting for 8.92 %. On multivariable logistic regression analysis, the factors associated with maternal mortality were: primary or no education (OR 1.9; 95 % CI, 1.0–3.3); HIV positive sero-status (OR, 3.6; 95 % CI, 1.9–7.0); no antenatal care attendance (OR 3.6; 95 % CI, 1.8–7.0); rural dwellers (OR, 4.5; 95 % CI, 2.5–8.3); having been referred from another health facility (OR 5.0; 95 % CI, 2.9–10.0); delay to seek health care (delay-1) (OR 36.9; 95 % CI, 16.2–84.4).

**Conclusions:**

Most maternal deaths occur among mothers from rural areas, uneducated, HIV positive, unbooked mothers (lack of antenatal care), referred mothers in critical conditions and mothers delaying to seek health care. Puerperal sepsis is the leading cause of maternal deaths at Mbarara Regional Referral Hospital. Therefore more research into puerperal sepsis to describe the microbiology and epidemiology of sepsis is recommended.

## Background

Maternal death is the death of a woman while she is pregnant or within 42 days of termination of pregnancy, irrespective of the duration and sites of the pregnancy, for any cause related to or aggravated by the pregnancy or its management, but not from accidental or incidental causes. Direct maternal death is the death of a woman resulting from obstetric complications of pregnancy, labor and puerperium; from interventions, omissions or incorrect treatment; or from a chain of events resulting from any of the above while indirect maternal death is the death of a woman resulting from a previously existing disease or a disease that developed during pregnancy and was not due to direct obstetric causes but was aggravated by the physiological effects of pregnancy [1, 2]. The global Maternal Mortality Ratio (MMR) is 210. [3, 4]. Comparably, the MMR reported for low resource settings such as sub-Saharan Africa is 500 while the developed countries, the rates are 16 maternal deaths per 100,000 live births. Despite global reduction in the MMR since the year 1990; the MMR is 15 times higher in low- resource countries than the resource rich countries [5, 6].

Low resource countries account for 99 % (286,000) of the global maternal mortalities with sub- Saharan Africa responsible for the bulk of the maternal deaths and accounting for 62 % followed by southern Asia at 24 %. In Uganda, there has been a slow decline in maternal mortality ratio (MMR) between 1990 and 2010 (from 550 in 1990 to 438 in 2012). Almost half of deliveries (52 %) in Uganda occur in health facilities and 59 % of all deliveries are assisted by a skilled birth attendant. The percentage of skilled attendance at birth has risen from 42 % to at least 59 % over the last 10 years. About 47 % of women attend at least 4 Antenatal Care (ANC) visits: while the adolescent birth rate is 134.5/1,000 births while the ANC HIV prevalence rate stands at 6.5 % [7, 8].

The causes of mortality vary and predictors of these maternal deaths in the Ugandan setting are largely unknown. For every woman who dies, about 30 women develop obstetrical near misses [9, 10]. Programs focusing on increasing health facility deliveries need to ensure that pregnant woman within the facility have quality health care during the antepartum, intrapartum and postpartum periods. Some of these interventions include improving on the understanding, attitudes and skills of the health care providers, improved involvement of the women in quality of care processes, ensuring implementation of evidence-based care and improving the referral systems [11].

Thaddeus Sereen and Deborah Maine in 1994 described three delays: Delay one (Delay to decide to make a decision to find health service), second delay (Delay to get to care) and third delay (Delay to access health care when at the health facility). For every maternal death that occurs, there is a one or more delays implicated. Many of these maternal deaths are preventable [12]. HIV/AIDS has become a major contributor to mortality especially where its prevalence is high.

Residence affects maternal health outcomes and may be associated with access to better learning opportunities, better financial status, better birth spacing, good accessibility to relevant information and health services, independence of decision making by the women, [13]. The education of the mother improves the mother’s socio-status, enhances their capability to make autonomous decisions and has been found to be a facilitator for improved health care access and a factor that enhances women’s ability to deliver from a health facility [14, 15].

Knowledge of the common causes and factors associated with mortality and morbidity in these women in our setting will help us tailor our preventive and treatment measures so as to be able to contribute towards improved maternal health outcomes. The aim of the study was to find out the causes and factors that influence the death of mothers at Mbarara Regional Referral Hospital (MRRH).

## Methods

### Design

It was a retrospective unmatched case control study.

### Setting

It was carried out on maternity ward of Mbarara Regional Referral Hospital, a public hospital found in Mbarara District. The Hospital registers about 12,000 deliveries annually and has cesarean section rate of 40 %.

### Selection and definition of cases

The cases were maternal deaths and these were retrospectively collected from the charts of mothers who died on maternity ward and qualified as maternal deaths.

### Selection and definition of controls

Controls were mothers who were admitted to the Maternity ward at MRRH for pregnancy related consultations such as abortion care, ectopic pregnancy managevment, vaginal and operative delivery care, Antepartum complications, and Post-delivery complications during the period January 2010-November 2014. The controls were mothers who did not die but survived and were discharged home alive. Information was retrospectively obtained from charts of these mothers. The charts were randomly selected.

### Sample size and power calculation

The sample sizes calculated for the study were 139 for the cases and 417 for the controls and these were adequate to achieve an 80 % power [16–20].

### Study variables

#### Dependent variable

The dependent variable (primary outcome) was maternal death. Direct obstetric death variables included obstetrical hemorrhage (antepartum hemorrhage and postpartum hemorrhage), postpartum sepsis, spontaneous or induced abortion, hypertensive diseases, obstructed labor, ruptured uterus, embolism and anesthetic complications. The Indirect obstetric death variables included, but not limited to, anemia, HIV, cardiac diseases, diabetes, renal disease, and malaria.

### Independent variables

The independent variables included the following: Age in years; Educational status (Post-primary or Primary/no education); Residence type; Referral status (referred or Not referred); Parity (Prime para-those who have delivered once, multi-para- between 2 and 4 deliveries, Grand-multipara- > 5 deliveries); HIV sero-status (Positive or negative); The three delays. Data was collected from these variables for a period of four (4) months by the research team.

### Statistical analysis

The data was analyzed using SPSS statistical software, version 20 (SPSS, Chicago, IL, USA). Cross tabulations conducted to obtain descriptive statistics were presented as frequencies, percentages and Pearson Chi-square statistics. Variables with *p*-value <0.1 at bivariable analysis were included in multivariable model. Odds ratios and the corresponding 95 % Confidence Intervals were presented and significance was accepted at *p* < 0.05.

### Quality assessment of the data

An independent Obstetrician, not part of the research team checked the completed data forms.

### Study limitations

The information collected was hospital data and therefore detailed community factors would not easily be assessed as no verbal autopsy was done on these particular cases. The data was also retrospective and therefore verification of certain aspects of data was difficult since we relied on information found in the charts, some of which was not of very good quality. Information about the age group with one of the highest risk of pregnancy complications and poor obstetrical outcomes (10–14 years) was not collected and analyzed [4].

### Ethical clearance

Was obtained ethical clearance from department of obstetrics and gynecology and Mbarara University of Science and Technology Institutional Research Committee.

## Results

### Causes of maternal deaths

Puerperal sepsis (30.9 %) was the leading cause followed by post- partum hemorrhage (21.6 %). The abortion complications included post-abortion septic shock and hemorrhage and these constituted 10.8 % while hypertensive disorders contributed 14.4 %. Majority of the mothers with hypertensive disorders had eclampsia and pre-eclampsia. The commonest cause of indirect maternal deaths was malaria contributing 40 % of all indirect maternal deaths. This is shown in Table 1.

**Table 1:** Causes of Maternal Mortality at Mbarara Regional Referral Hospital

Cause of death	Number (%)
Direct	
Puerperal sepsis	43 (30.9)
Hemorrhage	30 (21.6)
Abortion complications	15 (10.8)
Hypertensive diseases in pregnancy	20 (14.4)
Indirect causes	
Severe malaria	12 (8.92)
Anemia	06 (4.46)
Others	12 (8.92)

### Socio-demographic profiles and bivariable logistic regression

Majority of the women were married (82.7 % and 81.5 % respectively), the proportion of women among the cases who had attained primary or no education were higher at 61.9 % compared to the controls (31.9 %). Majority of the women constituting the controls were urban dwellers (65.5 %) compared to only 21.6 % among the cases. About 64 % of the cases were referred while only 21.8 % were referred among the controls; the HIV rates were higher in the cases compared to the controls (34.5 and 15.6 % respectively). In both the cases and the controls, the rates of the first delay were high (94.2 and 72.2 %) while delay-2 (delay to reach health care) was highest in the cases compared to the controls (77 and 38.8 % respectively) (Tables 2).

**Table 2:** Socio-demographic profiles and bivariable logistic regression

Variable	Cases n(%)	Controls n(%)	COR (95 % CI)	*p*-value
Age				
a) 15–24	50 (36.0)	203 (48.7)	1.9 (0.9–3.8)	0.079
b) 25–34	71 (51.1)	177 (42.4)	reference	
c) >34	34 (12.9)	37 (8.9)	2.8 (0.9–9.5)	0.089
Education level				
a) Post-Primary	53 (38.1)	254 (60.9)	reference	0.085
b) Primary or no education	86 (61.9)	163 (39.1)	1.7 (0.9–3.1)	
Residence type				
a) Urban	30 (21.6)	273 (65.5)	reference	<0.001
b) Rural	109 (78.4)	144 (34.5)	4.5 (2.4–8.3)	
Marital status				
a) Married	115 (82.7)	340 (81.5)	reference	0.998
b) Not Married	24 (17.3)	77 (18.5)	1.0 (0.4–2.4)	
Referral status				
a) No	50 (36)	326 (78.2)	reference	<0.001
b) Yes	89 (64)	91 (21.8)	5.4 (2.9–10.0)	
HIV sero-status				
a) Negative	91 (65.5)	352 (84.4)	reference	0.001
b) Positive	48 (34.5)	65 (15.6)	3.4 (1.6–6.9)	
Parity				
a) Primi-para	45 (32.4)	132 (31.7)	reference	
b) Multi-para	57 (41.0)	168 (40.3)	1.2 (0.5–2.4)	0.712
c) Grand Multi-para	37 (26.6)	117 (28.1)	0.6 (0.2–1.4)	0.212
Antenatal Care attendance				
a) Yes	79 (56.8)	318 (76.3)	reference	<0.001
b) No	60 (43.2)	99 (23.7)	3.6 (1.8–7.0)	
Delays				
a) Delay-1				
Yes	8 (5.8)	116 (27.8)	32.6 (13.5–78.6)	<0.001
No	131 (94.2)	301 (72.2)	Reference	
b) Delay-2				
Yes	32 (23) 107 (77)	255 (61.2)	1.4 (0.7–2.8)	0.281
No		162 (38.8)	reference	
c) Delay-3				
Yes	81 (58.3)	293 (70.3)	0.9 (0.5–1.7)	0.811
No	58 (41.7)	124 (29.7)	reference	

## Discussion

### Causes of maternal death

In our study, the direct causes contributed 77.7 % while the indirect for the remaining 22.3 %. Puerperal sepsis (30.9 %) was the leading cause followed by post- partum hemorrhage (21.6 %). This is in contrast with results from other studies and reports that show postpartum hemorrhage as the commonest cause of maternal deaths globally [21]. This difference is probably due to the fact that antibiotics are readily available for use in mothers that need them for prophylaxis and treatment to prevent sepsis while in our setting, the availability of such antibiotics is erratic and therefore the advantage conferred upon the mothers by the antibiotics is lost. Even when antibiotics are available, sepsis remains one of the commonest direct causes of death. Infections are gaining the first place in countries that had bleeding as the leading cause such as in Uganda and also in other countries where hypertensive disorders were the leading cause of maternal mortality. This seems to be a trend, not only for the ease in obtaining or not of antibiotics but by improvement in the handling of cases of hemorrhage and hypertension, which were the focus of maternal mortality over the last 15 years.

Obstetric hemorrhage is responsible for 27 % of the global causes of maternal deaths while sepsis accounts for 11 % [1]. In a related study at Mulago Hospital, obstetric hemorrhage and puerperal sepsis contributing 30.9 and 12.8 % respectively [22].

Severe pre-eclampsia/eclampsia and abortion complications (post abortion sepsis and hemorrhage) accounted for 14.4 and 10.8 % of deaths respectively. This is comparable to the WHO 2014 maternal mortality report by Say L which found that hypertensive disorders in pregnancy and abortion complications accounted for 14 and 8 % respectively [6]. This could be due to the fact that pregnancy induces similar physiological changes in all women irrespective of location and habitation and that complications arising from hypertensive disorders of pregnancy contribute to poor outcomes both in the low and well-resourced countries.

The commonest indirect cause of death was malaria which caused 8.92 % of the overall total deaths. The south western region of Uganda is endemic for malaria and this could explain the high mortality associated with malaria. This is however lower than rates reported in other Countries such as Nigeria where a country report by JHPIEGO, 2013 stated that malaria accounts for 11 % of all the country’s maternal death causes. This could be explained by the possibility of having more resistant falciparum malaria strains in West African Countries like Nigeria compared to Uganda and also the ready availability of antimalarial drugs for treating of malaria.

### Factors associated with maternal deaths

In our study, HIV positive women had a higher risk of dying compared to their HIV negative counterparts. This is comparable to other studies done elsewhere such as the study done in Mulago National Referral Hospital to evaluate the independent predictors of maternal mortality where they found that HIV/AIDS infection was predictive of maternal mortality (OR, 5.1; 95 % CI 2–12.8). A study in Rakai by Sewankambo et al, 2000 also found 5.4 odds of dying in HIV positive mothers compared to those who were HIV uninfected. The rate of HIV infection in the cases was higher than in the controls (34.5 and 15.6 % respectively). HIV infection has also been found to impact on pregnancy related outcomes. HIV positive women are eight times more likely to have a maternal death compared to HIV negative women [23, 21]. It is possible that HIV/AIDS lowers the body’s immunity and therefore the body’s ability to fight off opportunistic infections (Table 3).

**Table 3:** Multivariable Logistic Regression table showing factors associated with maternal deaths

Variable	Cases n(%)	Controls n(%)	cOR (95 % CI)	aOR (95 % CI)	*p*-value
Rural dwellers	109 (78.4)	144 (34.5)	4.5 (2.4–8.3)	4.5 (2.5–8.3)	<0.001
Being referred	89 (64)	91 (21.8)	5.4 (2.9–10.0)	5.0 (2.8–9.2)	<0.001
Primary or no education	86 (61.9)	163 (39.1)	1.7 (0.9–3.1)	1.9 (1.0–3.3)	0.037
No Antenatal Care (ANC) attendance	60 (43.2)	99 (23.7)	3.6 (1.8–7.0)	3.6 (1.9–6.9)	<0.001
Delay to decide to seek health care (delay 1)	131 (94.2)	301 (72.2)	32.6 (13.5–78.6)	36.9 (16.2–84.4)	<0.001
HIV positive sero-status	48 (34.5)	65 (15.6)	3.4 (1.6–6.9)	3.6 (1.9–7.0)	<0.001

It was also found out that lack of antenatal care increased the risk of maternal death (OR, 3.6; 95 % CI 1.8–6.9). Lack of antenatal care has been found in other studies as a predictor of maternal death (OR, 4.0; 95 % CI 1.3–9.2) [22]. Mothers who lack antenatal screening are predisposed to poor maternal obstetrical outcomes because they present late in very critical states with complications. Women in low resource settings are likely to miss out on important antepartum care schedules due to difficulties in accessing the health facilities.

Death was higher in women with less than post-primary education (OR, 2.3; 95 % CI, 1.2–4.2). The relationship between level of education and obstetrical outcomes has correspondingly been found in other studies. Educational level of women especially in low resource countries still remains a challenge [24, 25]. It is probable that the higher the level of education, the higher the opportunity of learning the health risks and therefore present for early screening. This helps to identify pregnancy complications early and therefore make strategies for minimizing adverse outcomes in mothers who present early and more regularly for antenatal care.

Being referred was from the peripheral health facilities was associated with a higher risk of dying (OR, 5.8; 95 % CI, 3.0–11.0). Other studies have showed that patients who are referred from lower health facilities are usually in critical states and their outcomes are poorer as compared to those not referred. [26]. Being referred from a peripheral health facility in critical condition is associated with poor obstetric outcomes, including mortality [19, 20].

The odds of death among mothers who were rural dwellers were higher compared to those who were staying in urban areas (OR, 3.8; 95 % CI, 2.0–7.0). There is a difference in maternal health outcomes as among rural dwellers compared to urban dwellers. Residence affects maternal health outcomes and urban residence may be associated with access to better learning opportunities, better financial status, better birth spacing, better accessibility to relevant information and health services, independence of decision making by the women, [13].

Mothers who delayed to seek health care had a higher risk of death (OR, 37.8; 95 % CI, 15.3–93.4). Some studies have found out that the delay to make a decision to seek care significantly impacts on health outcomes. In a study in Liberia, one of the factors found was self-treatment and failure to seek formal health care assistance and instead relying on traditional remedies [27].

## Conclusions

Most maternal deaths are seen in patients who are rural dwellers, have less than post primary education, HIV positive, referred and delay to seek health care. Puerperal sepsis is the commonest cause of maternal deaths. Delay to seek care is a very important factor contributing to poor obstetrical outcomes. HIV/AIDS has become a major contributor to mortality especially where its prevalence is high.

## Abbreviations

AIDS, Acquired Immune deficiency syndrome; CI, Confidence Interval; HIV, Human Immuno-deficiency virus; MMR, maternal mortality ratio; MRRH, Mbarara Regional Referral Hospital; MUST, Mbarara University of Science and Technology; OR, Odds Ratio; WHO, World Health Organization

## References

[1] World Health Organization, World Bank, UNICEF, UNFPA, United Nations Population Division: Trends in Maternal Mortality between 1990-2013.

[2] Ronsmans C, Walraven G, Etard JF (2004). Verbal autopsies: learning from reviewing deaths in the community. Beyond the Numbers. Reviewing Maternal Deaths and Complications to Make Pregnancy Safer.

[3] WHO, UNICEF, UNFPA, World Bank (2012). Trends in maternal mortality: 1990 to 2010.

[4] Oliveira FC, Surita FG, Pinto E, Silva JL, Cecatti JG, Parpinelli MA, Haddad SM, Costa ML, Pacagnella RC, Sousa MH, Souza JP (2014). Severe maternal morbidity and maternal near miss in the extremes of reproductive age: results from a national cross- sectional multicenter study. Brazilian Network for Surveillance of Severe Maternal Morbidity Study Group. BMC Pregnancy Childbirth.

[5] United Nations (2012). The Millennium Development Goals Report 2012.

[6] Say L, Chou D, Gemmill A, Tunçalp O, Moller AB, Daniels J, Gülmezoglu AM, Temmerman M, Alkema L (2014). Global causes of maternal death: a WHO systematic analysis. Lancet Glob Health.

[7] Uganda Bureau of Statistics. Uganda Demographic and Health Survey 2011. Uganda: Uganda Bureau of Statistics Kampala; 2012.

[8] Uganda Bureau of Statistics: Uganda Demographic and Health Survey 2006. Uganda: Uganda Bureau of Statistics Kampala; 2006.

[9] Ebuehi OM, Chinda GN, Sotunde OM, Oyetoyan SA (2013). Emergency Obstetric Care: Urban Versus Rural Comparison of Health Workers’ Knowledge, Attitude and Practice in River State, Nigeria. Implications for Maternal Health Care in Rivers State. Clin Med Diagn.

[10] Mukasa PK, Kabakyenga J, Senkungu JK, Ngonzi J, Kyalimpa M, Roosmalen VJ (2013). Uterine rupture in a teaching hospital in Mbarara, western Uganda, Unmatched case- control study. Reprod Health.

[11] Mathai M (2011). To ensure maternal mortality is reduced, quality of care needs to be monitored and improved alongside increasing skilled delivery coverage rates. BJOG.

[12] Thaddeus S, Maine D (1994). Too far to walk: Maternal Mortality in context. Sm Sci Med.

[13] Gabrysch S, Campbell OM (2009). Still too far to walk: literature review of the determinants of delivery service use. BMC Pregnanc Childbirth.

[14] Yalem T, Tesfay G, Isabel G, Kerstin E, Haile ML, Miguel SS (2013). Determinants of antenatal and delivery care utilization in Tigray region, Ethiopia: a cross-sectional study. Int J Equity Health.

[15] Woldemichael G, Tenorang EY (2010). Women’s autonomy and maternal health seeking behavior in Ethiopia. Matern Child Health J.

[16] Chow SC, Shao J, Wang H (2003). Sample Size Calculations in Clinical Research.

[17] D’Agostino RB, Chase W, Belanger A (1988). The Appropriateness of Some Common Procedures for Testing the Equality of Two Independent Binomial Populations. Am Stat.

[18] Fleiss JL, Levin B, Paik MC (2003). Statistical Methods for Rates and Proportions.

[19] Lachin JM (2000). Biostatistical Methods.

[20] Machin D, Campbell M, Fayers P, Pinol A (1997). Sample Size Tables for Clinical Studies.

[21] Carine R, Wendy JG (2006). Maternal mortality: who, when, where, and why. The Lancet Maternal Survival Series steering group. Lancet.

[22] Julius NW, Pat D, Benjamin LM, Paul K, Betty K, Emmanuel O, Noreen M (2011). Human immunodeficiency virus and AIDS and other important predictors of maternal mortality in Mulago Hospital Complex Kampala Uganda. BMC Public Health.

[23] Calvert C, Ronsmans C, The contribution of HIV to pregnancy-related mortality: a systematic review and meta-analysis. AIDS 27, advance online edition, doi: 10.1097/qad.0b013e32835fd940, 2013 10.1097/QAD.0b013e32835fd940 PMC3678884 23435296

[24] Alvarez JL, Gil R, Hernández V, Gil A (2009). Factors associated with maternal mortality in Sub-Saharan Africa: an ecological study. BMC Public Health.

[25] Yego F, D’Este C, Byles J, Williams JS, Nyongesa P (2014). Risk factors for maternal mortality in a Tertiary Hospital in Kenya: a case control study. BMC Pregnancy and Childbirth.

[26] Kongnyuy EJ, Mlava G, van den Broek N (2009). Facility-based maternal death review in three districts in the central region of Malawi: an analysis of causes and characteristics of maternal deaths. Womens Health Issues.

[27] J R Lori, A E. Starke. A critical analysis of maternal morbidity and mortality in Liberia, West Africa: Midwifery 2011. doi:10.1016/j.midw.2010.12.001 10.1016/j.midw.2010.12.001 21232836

